# QTL Mapping Combined With Comparative Analyses Identified Candidate Genes for Reduced Shattering in *Setaria italica*

**DOI:** 10.3389/fpls.2018.00918

**Published:** 2018-07-19

**Authors:** Sandra Odonkor, Soyeon Choi, Debkanta Chakraborty, Liliam Martinez-Bello, Xuewen Wang, Bochra A. Bahri, Maud I. Tenaillon, Olivier Panaud, Katrien M. Devos

**Affiliations:** ^1^Institute of Plant Breeding, Genetics and Genomics, University of Georgia, Athens, GA, United States; ^2^Department of Genetics, University of Georgia, Athens, GA, United States; ^3^Institute of Bioinformatics, University of Georgia, Athens, GA, United States; ^4^Department of Plant Biology, University of Georgia, Athens, GA, United States; ^5^Laboratory of Bioagressors and Integrated Protection in Agriculture (LR14AGR02), The National Agronomic Institute of Tunisia, University of Carthage, Tunis, Tunisia; ^6^UMR8120 Génétique Quantitative et Evolution Le Moulon, Institut National de la Recherche Agronomique, Université Paris-Sud, Centre National de la Recherche Scientifique, AgroParisTech, Université Paris-Saclay, Paris, France; ^7^Laboratoire Génome et Développement des Plantes, UMR UPVD/CNRS, Université de Perpignan Via Domitia, Perpignan, France

**Keywords:** domestication, miniature inverted-repeat transposable element (MITE), QTL analysis, seed shattering, *Setaria italica*, *Setaria viridis*, transcription factors

## Abstract

*Setaria* (L.) P. Beauv is a genus of grasses that belongs to the *Poaceae* (grass) family, subfamily Panicoideae. Two members of the *Setaria* genus, *Setaria italica* (foxtail millet) and *S. viridis* (green foxtail), have been studied extensively over the past few years as model species for C4-photosynthesis and to facilitate genome studies in complex Panicoid bioenergy grasses. We exploited the available genetic and genomic resources for *S. italica* and its wild progenitor, *S. viridis*, to study the genetic basis of seed shattering. Reduced shattering is a key trait that underwent positive selection during domestication. Phenotyping of F_2:3_ and recombinant inbred line (RIL) populations generated from a cross between *S. italica* accession B100 and *S. viridis* accession A10 identified the presence of additive main effect quantitative trait loci (QTL) on chromosomes V and IX. As expected, enhanced seed shattering was contributed by the wild *S. viridis*. Comparative analyses pinpointed *Sh1* and *qSH1*, two shattering genes previously identified in sorghum and rice, as potentially underlying the QTL on *Setaria* chromosomes IX and V, respectively. The *Sh1* allele in *S. italica* was shown to carry a *PIF/Harbinger* MITE in exon 2, which gave rise to an alternatively spliced transcript that lacked exon 2. This MITE was universally present in *S. italica* accessions around the world and absent from the *S. viridis* germplasm tested, strongly suggesting a single origin of foxtail millet domestication. The *qSH1* gene carried two MITEs in the 5′UTR. Presence of one or both MITEs was strongly associated with cultivated germplasm. If the MITE insertion(s) in *qSH1* played a role in reducing shattering in *S. italica* accessions, selection for the variants likely occurred after the domestication of foxtail millet.

## Introduction

*Setaria italica*, foxtail millet, and its wild ancestor *Setaria viridis*, green foxtail, are two of the ∼100 species that comprise the genus *Setaria*, tribe Paniceae, subtribe Cenchrinae, subfamily Panicoideae (Kellogg, 2017). The cultivated foxtail millet is grown both as a grain crop and for forage. The wild *S. viridis* is a weed found around the world in disturbed habitats. Cultivation of *S. italica* began approximately 5,900 years ago in the northern region of China, making it one of the oldest known domesticated crops (Barton et al., 2009). Domestication is the result of convergent selection for a limited number of traits aimed at making the crop better adapted to agricultural environments, higher yielding, and easier to harvest. The main traits favored in domesticated cereals are non-shattering seeds, larger grain size, increased grain number, larger panicle size, higher grain quality, and reduced sensitivity to photoperiod (Gepts, 2010).

Over the past decade, genes involved in a number of traits targeted by domestication have been characterized in cereal crops. One of the first genes isolated underlying a domestication quantitative trait locus was *Teosinte-branched* (*Tb1*) in maize, a transcription factor that controls axillary branch formation, sex expression, and inflorescence architecture (Doebley et al., 1997). Transition from the highly branched maize ancestor, teosinte, to the single-stalk phenotype of cultivated maize was caused by increased expression of *Tb1*, effected by the insertion of a transposable element 63 kb upstream of *Tb1*. Transcription factors have also been shown to be involved in the control of seed shattering in cereals (Doebley, 2006). *SH4* and *qSH1* are the two major disarticulation genes that underwent selection during rice domestication. *SH4*, located on rice chromosome 4, is a member of the trihelix family of transcription factors and carries a Myb3 DNA binding domain (Li et al., 2006). A single nucleotide polymorphism (SNP) in the Myb3 domain greatly reduced shattering. More recently, a frameshift mutation in *SH4* was identified that completely eliminated shattering (Zhou et al., 2012). *qSH1* on rice chromosome 1 encodes a BEL1-type transcription factor (Konishi et al., 2006). A SNP in the 5′ regulatory region eliminated *qSH1* expression at the abscission layer leading to loss of shattering ability. In sorghum, selection for non-shattering phenotypes during domestication largely occurred at the *Sh1* locus on chromosome 1 (Lin et al., 2012). At least three independent mutations have been identified that either inactivate the gene or reduce its expression significantly. Syntenic regions on rice chromosome 3, maize chromosomes 1 and 5, and *Setaria* chromosome IX have been shown to harbor disarticulation QTL, suggesting parallel selection at the *Sh1* locus during the domestication of cereals (Lin et al., 2012). Free-threshing ability in wheat is conferred by mutations in the *Q* gene on wheat chromosome 5A, which encodes a transcription factor belonging to the APETALA2 family. Wild and cultivated alleles differ in their expression as well as by the presence of a non-synonymous SNP which causes reduced homodimer formation (Simons et al., 2006). Triticeae also display disarticulation of the rachis, which is controlled by two tightly linked genes on chromosome 3 (Komatsuda et al., 2007). Inactivation of one of the two genes is sufficient to yield a non-brittle rachis phenotype. One of the genes (*BTR1*) is predicted to encode a membrane-bound protein. The other gene (*BTR2*) likely encodes a soluble protein. BTR2 has some similarity to, but is not considered an ortholog of, the *Arabidopsis thaliana INFLORESCENCE DEFICIENT IN ABSCISSION* (Butenko et al., 2003).

*Setaria* has recently been brought to the spotlight as a model plant system to study agronomically relevant traits in C_4_-photosynthetic biofuel grasses and cereals (Doust et al., 2009; Li and Brutnell, 2011). *S. italica* and *S. viridis* have small diploid genomes (∼450 Mb), a short stature, rapid life cycle, and prolific seed production. Genetic and genomic resources include whole-genome sequence assemblies for both *S. italica* (Bennetzen et al., 2012; Zhang et al., 2012) and *S. viridis* (*Setaria viridis* v1.1, DOE-JGI^1^), a recombinant inbred line (RIL) population derived from a cross between B100 (*S. italica*) and A10 (*S. viridis*) (Bennetzen et al., 2012), high-density genetic maps (Bennetzen et al., 2012; Fang et al., 2016; Wang et al., 2017), a high-density haplotype map of genome variation (Jia et al., 2013) and transformation capability (Van Eck et al., 2017). QTL for seed shattering have previously been reported on *Setaria* chromosomes V and IX (Devos and Gale, 2000; Doust et al., 2014a). Here, we exploit the available genetic and genomic resources to further determine the genetic basis of shattering loss in foxtail millet.

## Materials and Methods

### Plant Material

Seed shattering was measured on F_2:3_ progeny (Devos et al., 1998; Wang et al., 1998) as well as on F_10_ recombinant inbred lines (RIL) (Bennetzen et al., 2012) from a cross between *S. italica* accession B100 and *S. viridis* accession A10. A total of 112 F_2:3_ families of 6–13 plants together with the parents were grown in a randomized block design with two replications during the period from May to September 1998 in the field at Orsay, France. Replicate 2 consisted of only 66 F_2:3_ families because of insufficient seed for some of the lines. For the RIL population, four plants per line for a total of 188 lines were grown in hill plots in a randomized block design with three replications at the University of Georgia (UGA) Plant Sciences Farm, Watkinsville, GA, during the period June to August, 2011. The *S. italica* accessions Yugu1 and B100 and the *S. viridis* accession A10 were part of the UGA *Setaria* collection. Their sources have been described previously (Wang et al., 1998; Bennetzen et al., 2012). *S. italica* accessions Ise-3 and Ise-5 were obtained from ICRISAT, Patancheru.

### Genetic Maps and Markers

A linkage map for the F_2_ population had been developed by Wang and colleagues using restriction fragment length polymorphism (RFLP) markers (Devos et al., 1998; Wang et al., 1998). This map consisted of 257 loci spanning 1050 cM across the nine foxtail millet chromosomes. The genetic map generated in the RIL population and reported by Bennetzen et al. (2012) consisted of 990 single nucleotide polymorphism (SNP) markers and comprised a total map length of 1416 cM. The genotypic data used for the generation of both maps are attached as **Supplementary Table S1**.

### Phenotypic Evaluation of Traits

Shattering ability, that is the ease with which spikelets are released from the pedicel, was scored subjectively from 0 (florets remaining intact as in the cultivated *S. italica*) to 3 (freely shattering as in the wild *S. viridis*) by clasping the panicle in a clenched fist. The phenotypic data used in the QTL mapping are provided in **Supplementary Table S1**.

### QTL Mapping and Candidate Gene Identification

QTL mapping in both the F_2:3_ and RIL populations was done using composite interval mapping (CIM) in Windows QTL Cartographer 2.5 (Basten et al., 1995; Wang et al., 2012). We used model 6 with forward and backward regression, a 10 cM window size, and a mapping step size of 1 cM. The logarithm of odds (LOD) threshold that defined a significant QTL was determined based on 500 permutations and a significance level of *P* = 0.05. Analyses were done for each replicate separately, as well as across replicates. To find candidate genes underlying the QTL, QTL regions, delineated by the most distal flanking markers with LOD scores above the significance threshold, were located on the *S. italica* Yugu1 genome sequence assembly v2.2 ^2^. Using comparative knowledge between *Setaria* and other grass species (Bennetzen et al., 2012; Devos et al., 2017), the orthologous regions in rice, sorghum, and maize were scanned for the presence of known shattering genes.

### Comparative Analysis at the DNA Sequence Level of the *S. italica* and *S. viridis* Alleles at the *Sh1* and *qSH1* Loci

The sorghum *Sh1* (Sobic.001G152901; Lin et al., 2012) and rice *qSH1* (LOC_Os01g62920) coding sequences were used in BLASTN searches against the *S. italica* Yugu1 and *S. viridis* A10 whole genome sequence assemblies^2^ to identify the corresponding orthologs. For each gene, the genomic sequences of the *S. italica* and *S. viridis* alleles were aligned and compared. To determine whether the same structural variations were present in B100 as in Yugu1, we exploited the ∼43 Gb of sequence data that were available from a bulk of 48 B100 × A10 RIL lines (Bennetzen et al., 2012; SRX030717 and SRX030718). The RIL bulk sequencing data had been generated to identify the SNPs used in the construction of the RIL genetic map (Bennetzen et al., 2012). The Illumina reads (2 × 75 bp) were aligned against the Yugu1 genome assembly using Bowtie (Langmead and Salzberg, 2012) with default parameters. Alignments in the *Sh1* and *qSH1* regions were inspected visually using the Integrated genome viewer (IGV) v2.3 (Robinson et al., 2011).

The structural variants identified in *Setaria* in *Sh1* and *qSH1* were further validated by PCR-amplification using primer set SiSh1-91F (5′-GGATCATGCCTTGCACTCCT-3′) / SiSh1-91R (5′-CATGCATGCACATTTCGGCT-3′) for *Sh1* and qSHIns1_F (5′-GCTTCCTGGAGTGGTCAAAC-3′) / qSHIns1_R (5′-GGACGTGGTAGAGCTGCTG-3′) for *qSH1*, and genomic DNA of *S. italica* accessions Yugu1 and B100, and *S. viridis* accession A10. PCR reactions consisted of 1.5 mM MgCl_2_, 200 nM dNTPs, 0.5 μM of forward and reverse primer and 4 U of GoTaq DNA polymerase (Promega) in 50 μl 1X GoTaq buffer (Promega) either with or without 5% DMSO. PCR conditions for *Sh1* were initial denaturation at 95°C for 3 min, 40 cycles of denaturation at 95°C, annealing at 58°C for 40 s, ramping to 72°C at a rate of 1°C/s and extension at 72°C for 3 min, followed by a final extension of 5 min at 72°C after which reactions were held at 10°C. The same conditions were used for *qSH1* except that reaction volumes were decreased to 10 or 20 μl, annealing was done at 60.9°C, and the number of cycles was reduced to 35. Amplicons were gel extracted using the Zymoclean^TM^ Gel DNA Recovery Kit (The Epigenetics Company) and Sanger sequenced. Because of low amplification efficiency, *Sh1* fragments amplified from B100 were reamplified following gel extraction using the same reaction conditions, gel extracted again, and then sequenced.

### Assessing the Presence of the MITEs in *Sh1* and *qSH1* in *S. italica* and *S. viridis* Germplasm

We downloaded Illumina reads obtained from whole-genome resequencing of 200 *S. italica* accessions (Jia et al., 2013) and 100 *S. viridis* accessions ^3^ and used sequences that spanned the boundary between genic and MITE sequence, as well as genic sequence that flanked the MITE insertion sites (*S. viridis* control) as queries in BLASTN analyses to test for the presence of the MITEs in the sequence data. The absence of hits when the *S. viridis* control was used as query combined with the presence of hits when the boundary region between genic sequence and MITE was used as query was taken as evidence for the presence of the MITE.

### Transcript Analysis of *Sh1* and *qSH1* in *S. italica* and *S. viridis*

Total RNA was isolated from seedling leaves and from panicles 15 and 40 days after panicle emergence (15 DAPE) from A10 (*S. viridis*) and B100 (*S. italica*) using Trizol. Genomic DNA contamination was removed using the Turbo DNA*-free* kit (Invitrogen). cDNA was prepared using 500 ng of total RNA using the RevertAid First Strand cDNA Synthesis Kit (Thermo Scientific) according to the manufacturer’s instructions. To assess the effect of the MITE on *Sh1* transcript processing in *S. italica*, 2 μg of cDNA was used as template in a PCR reaction (total volume 25 μl) consisting of 2 mM MgCl_2_, 200 nM dNTPs, 1 μM forward primer Sh1-09f-MITE (5′-GTGCTACGTACACTGCAACTT-3′; located in exon 1), 1 μM reverse primer Sh1-09r-MITE (5′-ATGATCCTGATCGCCTTTTG-3′; located in exon 3), and 0.025 U of GoTaq (Promega) in 25 μl of GoTaq Reaction Buffer (Promega). Amplicons were checked on a 1% agarose gel, purified from the gel using the GeneJET Gel Extraction Kit (Thermo Scientific), cloned into the pGEM-T Easy Vector (Promega) and Sanger sequenced. Semi-quantitative (semi-q) RT-PCR was conducted to determine transcript levels of *qSH1* in A10 and B100 using primer sets qSH_expr1_F (Seq: 5′- TCCAACTGATGGTGACAAGC-3′) and qSH_expr1_R (Seq: 5′- CTTGTGGACCTGCCTCATCT-3′). Actin was used as control. Reaction conditions were the same as used for amplification of genomic DNA. The semi-qRT-PCR conditions for analyzing *qSH1* expression levels were initial denaturation at 95°C for 5 min, 30 cycles of denaturation at 95°C for 45 s, annealing at 60°C for 45 s, and extension at 72°C for 1 min, followed by a final extension of 5 min at 72°C after which reactions were held at 10°C. A total of 5 μl reaction product was run on a 2.0% agarose gel and the intensity of the bands was measured using the software ImageJ (Abràmoff et al., 2004). Band intensity for *qSH1* was normalized using the intensity of the actin amplicon. *qSH1/Actin* ratios of technical replicates were averaged for statistical analysis.

## Results

### Phenotypic Variation

Shattering in *S. viridis* acc. A10 was scored as 3 (highly shattering) and in *S. italica* acc. B100 as 0 (non-shattering). Some 39% of F_2:3_ families displayed little shattering (mean score ≤ 1.3) and 2% displayed high shattering (mean score ≥ 2.7). Similarly, in the RIL population, an average of 35% of RILs were low shattering and 5% were highly shattering.

### QTL Detection

**FIGURE 1:**
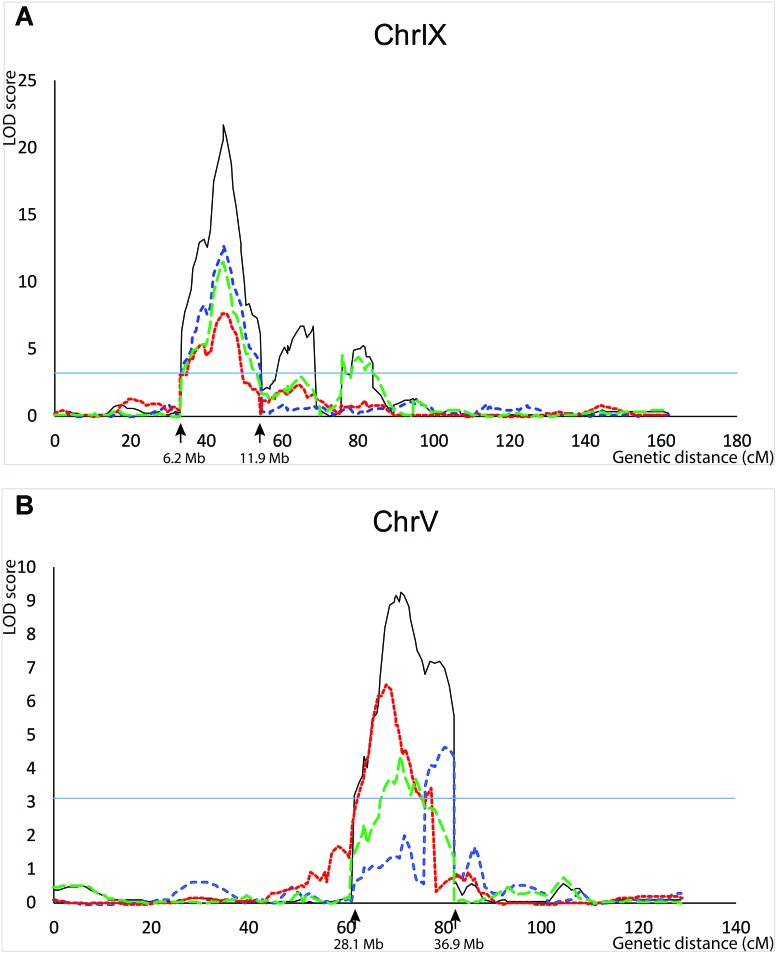
QTL identified in the B100 × A10 RIL population on chromosomes IX **(A)** and V **(B)**. QTL in replicate 1 are in blue, in replicate 2 in red, in replicate 3 in green, and across-replicates in black. The horizontal line indicates the LOD threshold for significance at the 5% level.

QTL for seed shattering were identified on chromosomes V, VI, and IX in the F_2:3_ population and on chromosomes V, VII, and IX in the RIL population (**Table 1**). For all QTL, the A10 allele led to increased shattering. The chromosome IX QTL in the F_2:3_ population was located between the rice cDNA sequences C0595 (Genbank accession number AU091626) and C1361 (C98269), which identify loci on the *S. italica* acc. Yugu1 genome sequence (Bennetzen et al., 2012) at positions 6.2 and 14.6 Mb, respectively. This QTL explained 30.6% of the variation in the across-replicate analysis. The main chromosome IX QTL in the RIL population was located in the region 6.2 Mb–11.9 Mb (**Figure 1A**) and explained 35.2% of the variation in the across-replicate analysis (**Table 1**). QTL on chromosome V were also identified in all replicates in both populations although the location of the QTL varied by replicate and population. The chromosome V QTL in the F_2:3_ population mapped to non-overlapping regions on the *S. italica* genome sequence (0.6–8.8 Mb and 29.5–to 43.7 Mb for replicate 1 and replicate 2, respectively). No QTL was identified on chromosome V when mean values across replicates 1 and 2 were used as trait data. In the RIL population, the chromosome V QTL was delineated to the region 35.8–36.9 Mb in replicate 1, 28.5–35.4 Mb in replicate 2, 32.3–35.4 Mb in replicate 3, and 28.1–36.9 Mb in the across-replicate analysis (**Figure 1B**). This QTL explained 12.5% of the variation in the across-replicate analysis (**Table 1**). The QTL on chromome VI was significant only in replicate 2 in the F_2:3_ population. Similarly, the QTL on chromosome VII was significant only in replicate 2 in the RIL population.

**Table 1:** QTL for seed shattering detected in the F_2:3_ and RIL populations.

	Replicate	Chromosome	Flanking Markers (Position in cM)	Marker at Peak (Position in cM)	LOD at Peak	Additive Effect	Dominant Effect	R^2^ (%)
F_2_ population^1^	Rep1	V	psf63.1 (37.9) – rgc643.1 (42)	psf63.1 (37.9)	5.7	−0.411	0.020	15.5
	Rep2	V	rgc409 (66.3) – psf386 (80.9)	rgc409 (66.3)	9.7	−0.499	0.015	35.4
	Rep2	VI	rgc83.4 (61.6) – psf420 (69.6)	psf420 (69.6)	5.0	−0.290	−0.050	9.7
	Rep1	IX	rgc1361 (100.2) – rgc595 (128.5)	rgc136 (124.5)	9.4	−0.306	0.393	29.0
	Rep2	IX	rgc136 (124.5) – rgc595 (128.5)	psm176 (127.6)	7.8	−0.444	−0.086	16.8
	All	IX	rgc1361 (100.2) – rgc595 (128.5)	rgc136 (124.5)	10.7	−0.374	0.259	30.6
RIL population^2^	Rep1	V	UGSF364 (76.2) – UGSF365 (78.2)	UGSF365 (78.2)	4.1	0.218	0	7.6
	Rep2	V	UGSF345 (63.4) – UGSF364 (76.2)	UGSF350 (66.9)	6.2	0.302	0	12.7
	Rep3	V	UGSF352 (70.0) – UGSF361 (74.7)	UGSF356 (71.3)	4.4	0.247	0	7.7
	All	V	UGSF343 (61.7) – UGSF365 (78.2)	UGSF356 (71.3)	9.3	0.253	0	12.5
	Rep2	VII	UGSF621 (12.9)	UGSF621 (12.9)	3.5	0.223	0	6.7
	Rep1	IX	UGSF21 (33.2) – UGSF39 (54.3)	UGSF31 (44.4)	12.7	0.395	0	24.5
	Rep2	IX	UGSF22 (34.7) – UGSF34 (48.9)	UGSF31 (44.4)	7.6	0.388	0	16.0
	Rep3	IX	UGSF21 (33.2) – UGSF38 (52.1)	UGSF30 (44.2)	11.5	0.489	0	22.3
	All	IX	UGSF21 (33.2) – UGSF39 (54.3)	UGSF31 (44.4)	21.8	0.481	0	35.2
	All	IX	UGSF48 (58.4) – UGSF83 (68.3)	UGSF54 (64.6)	6.7	−0.273	0	8.7
	Rep3	IX	UGSF96 (75.6) – UGSF109 (83.9)	UGSF96 (75.6)	4.5	0.317	0	7.9
	All	IX	UGSF96 (75.6) – UGSF108 (82.8)	UGSF103 (81.8)	5.1	0.242	0	6.5

*^1^B100 = Parent A; A10 = Parent B; LOD thresholds for QTL significance were 3.8 in Rep1, 3.7 in Rep2 and 3.8 across-replicates. ^2^A10 = Parent A; B100 = Parent B; LOD thresholds for QTL significance were 3.1 for Reps 1, 2 and across-replicates, and 3.0 for Rep 3.*

### Identification of Candidate Genes Underlying the Chromosome V and IX QTL

Several genes controlling seed shattering have been identified in cereals. Considering the known grass syntenic relationships (Gale and Devos, 1998; Devos, 2005), *qSH1* (Konishi et al., 2006) and *Sh1* (Lin et al., 2012) were of specific interest as candidate genes for the shattering QTL in our study. These genes had also been identified by Doust et al. (2014a,b) as candidates for shattering QTL they had mapped to *Setaria* chromosomes V and IX. *qSH1* is a BEL1-type homeobox gene on rice chromosome 1 which is syntenic with *Setaria* chromosome V. *Sh1* is a YABBY transcription factor on sorghum chromosome 1, which is syntenic with *Setaria* chromosome IX. The ortholog for *Sh1* in *S. italica* accession Yugu1 (Seita.9G154300) was located at position 10.1 Mb (corresponding to ∼44.5 cM on the genetic map) and thus within the interval carrying the shattering QTL on chromosome IX (6.2–11.9 Mb; **Figure 1A**). Furthermore, the marker most highly associated with the shattering trait on chromosome IX in the RIL population, UGSF31, was also the marker most closely linked to the *Sh1* gene. A comparison of the genomic and coding sequences of Seita.9G154300 and its ortholog in *S. viridis* acc. A10 (Sevir.9G153200) showed the presence of an 854 bp miniature inverted-repeat transposable element (MITE) in exon 2 in Seita.9G154300. The MITE belonged to the *P-Instability Factor (PIF)/Harbinger* family of DNA transposons. Scrutiny of this region in the alignment of Illumina reads generated from a pool of 48 B100 × A10 RILs showed the presence of reads that spanned the boundaries between *Sh1* and MITE sequence, suggesting that the MITE was also present in *S. italica* accession B100. PCR amplification of the MITE-containing region in *S. italica* accessions Yugu1 and B100 using primers located in intron 1 and intron 2 yielded no product using standard PCR conditions, and a faint ∼1500 bp product when amplification was done in the presence of 5% DMSO. In contrast, both sets of reaction conditions yielded a strong ∼700 bp product in *S. viridis* accession A10. Sequencing of the B100 amplicon showed that this accession carried the same MITE that was present in *S. italica* accession Yugu1 (**Supplementary Figure S1**).

The *Setaria* orthologs for *qSH1* were Seita.5G381300 in *S. italica* acc. Yugu1 and Sevir.5G386500 in *S. viridis* acc. A10. Seita.5G381300 maps to position 41.4 Mb (corresponding to ∼86 cM on the genetic map) in the *S. italica* Yugu1 genome assembly. Although none of the chromosome V QTL identified in our study spanned the *qSH1* locus (**Figure 1B**), we nevertheless considered *qSH1* as putatively underlying the shattering QTL on *Setaria* chromosome V because of its close proximity to the QTL. A comparison of Sevir.5G386500 and Seita.5G381300 showed that the two genes differed by a non-synonymous SNP in the coding region leading to a Q_77_ → H_77_ amino acid substitution, and by the presence of two MITEs belonging to the *PIF/Harbinger* family in the 5′UTR region. The insertion of both MITEs had been accompanied or followed by a rearrangement (**Supplementary Figure S2**). In case of the most 5′ located MITE (MITE 1), the deletion which spanned the 5′ boundary region of the *qSH1* 5′UTR and MITE was flanked by a short direct CGG repeat. This suggests that the deletion occurred by non-homologous end-joining during DNA break repair, possibly as part of the MITE insertion event. The insertion of the most 3′ located MITE (MITE 2) was accompanied/followed by both a deletion and an insertion (**Supplementary Figure S2**). Scrutiny of the alignment of the bulked B100 × A10 RIL reads against the Yugu1 genome assembly showed that the SNP and both MITEs were also present in *S. italica* accession B100. The presence of the two MITEs in the 5′UTR of B100 was confirmed by PCR and Sanger sequencing of the amplicons (**Supplementary Figure S3**). Semi-quantitative RT-PCR of *qSH1* in leaves of B100 and A10 showed that transcript levels were significantly lower in B100 compared to A10 (*P* = 0.015) (**Supplementary Table S2**). *qSH1* transcript levels in panicles were higher than in leaves (**Supplementary Table S2**), but not significantly different between A10 and B100 (*P* = 0.212).

### Effect of the MITE on Transcript Processing of *Sh1* in *S. italica*

**FIGURE 2:**
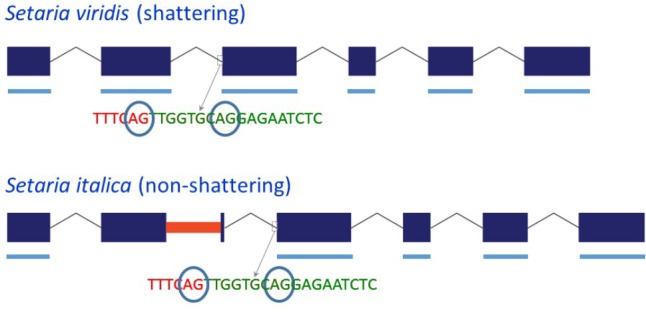
Schematic representation of the structure of the *Sh1* gene and its transcript in *S. viridis* and *S. italica*. Blue boxes represent exons and thin lines represent introns. The red bar in exon 2 represents the MITE insertion. Thick blue lines underneath the exons represent exonic sequence present in the transcript. The circled “AG” indicates alternative splice sites.

Because the MITE in Yugu1 and B100 *Sh1* was inserted close to the 3′ end of exon 2, we investigated whether its presence affected splicing. Sequencing of four clones from a single PCR reaction using *S. viridis* accession A10 cDNA as template with primer set sh1-09f-MITE/sh-09r-MITE, which was located in exon 1/exon 3 and hence amplified across the MITE insertion site in exon 2, indicated the presence of two potential 3′ splice sites for intron 2 that were separated by 9 bp (**Figure 2** and **Supplementary Figure S1**). Reads corresponding to both splice products were also identified by BLASTN analysis against *S. viridis* A10 transcriptome data (NCBI SRA Experiment SRX875196), supporting the occurrence of alternative splicing. Sequencing of the same region (two clones from the same PCR reaction) in a *S. italica* B100 transcript also showed two alternative splice products that differed by nine basepairs. Furthermore, B100 transcripts lacked exon 2 (**Figure 2** and **Supplementary Figure S1**). The presence of the MITE resulted in the simultaneous splicing of intron 1, exon 2 plus the MITE and intron 2. The transcript data obtained by RT-PCR were confirmed by alignment of available RNA-Seq data from *S. italica* Yugu1 (NCBI SRA Experiment SRX2832831) against Seita.9G154300. The alignments further suggested that the MITE presence led to an additional alternatively spliced product in which introns 1 and 2 were retained (**Supplementary Figure S4**). We were unable to confirm the formation of this putatively alternatively spliced product by RT-PCR with primers located in exon 2 and exon 3. This could be due to the difficulty in amplifying across the GC-rich MITE. The overall GC content of the MITE was 66%, but was as high as 90% in several 50 bp windows. Alternatively, the presence of introns 1 and 2 in the RNA-Seq data may have been caused by genomic DNA contamination. This, however, is unlikely, because no evidence was obtained of retention of introns 3, 4, and 5 (**Supplementary Figure S4**). Of the two alternative splice sites 9 bp apart between intron 2 and exon 3, use of only the most 5′ splice site was observed in the RNA-Seq reads.

### Presence of the MITEs in *S. italica* and *S. viridis* Germplasm

To determine the prevalence of the MITEs in *Sh1* and *qSH1* in *Setaria* germplasm, regions of, on average 87 bp, that covered either the boundary between genic sequence and MITE sequence or were comprised of genic sequence flanking the MITEs, were used in BLASTN searches against resequencing data from 200 *S. italica* and 100 *S. viridis* accessions. One or more reads in 59 *S. italica* accessions had hits against the boundary region that covered the 3′ end of exon 2 and the 5′ end of the MITE in *Sh1*. A total of 95 *S. italica* accessions had hits using the last 40 bp of the MITE, the last 5 bp of exon 2 and the first 39 bp of intron 2 of Yugu1 *Sh1* as query sequence. Thirty-five accessions had hits for both the 5′ and 3′ boundary regions, bringing the total number of *S. italica* accessions that carried a MITE to 119. None of the *S. italica* accessions had BLAST hits against the *S. viridis* control query sequence which covered the last 28 bp of exon 2 and the first 44 bp of intron 2 of A10 *Sh1*. In contrast, all 100 *S. viridis* accessions had BLAST hits using the MITE flanking sequence (*S. viridis* control) as query; none has BLAST hits using genic-MITE boundary regions as queries.

We found significant variation for the presence of the two MITEs in *qSH1* in *S. italica* germplasm. Of the 122 *S. italica* accessions with one or more identified blast hits, 54% carried at least one of the two MITEs, 43% lacked one of the MITEs but the presence of the other MITE was unknown and 2.5% (3 accessions) lacked both MITEs (**Supplementary Table S3**). Both MITEs were absent from the 5′UTR in *qSH1* in all *S. viridis* germplasm tested.

## Discussion

Although shattering ability was evaluated in a simple manner by scoring seed loss on a 0–3 scale, QTL were consistently (i.e., across replicates and populations) identified on chromosomes V and IX. However, only the QTL on chromosome IX colocalized across populations and replicates. This suggests that the clenched-fist method was sufficiently accurate to identify shattering loci of large effect. Precise mapping of smaller-effect QTL, however, may require the use of a force gauge to more precisely measure the strength needed to disarticulate a spikelet. The presence of wild-type alleles at both the chromosomes V and IX loci was required for a high level of shattering, indicating that the chromosomes V and IX effects are additive. Although the two QTL combined explained less than 50% of the variation in shattering, this is likely an underestimation due to the imprecise phenotyping. Nevertheless, it is possible that smaller-effect QTL for shattering are present in the population which were not, or not consistently, detected. The QTL on chromosomes VI and VII which were identified in only one replicate in the F_2:3_ population and RIL population, respectively, could be such examples. Furthermore, additional QTL peaks were identified in some replicates on chromosome IX, and it is possible that they represent additional shattering genes that are present in the B100 × A10 population.

Although no visual abscission layer is present in *Setaria* (Hodge and Kellogg, 2016), our data show that the function of *Sh1* in seed disarticulation is conserved with *Sh1* function in other grass species despite differences in structure and/or location of the abscission zone (Doust et al., 2014b). The YABBY transcription factor (*Sh1* gene) underlying the shattering QTL on chromosome IX has previously been identified as the main gene controlling disarticulation in domesticated sorghum (Lin et al., 2012). *Sh1* also underlies shattering QTL on rice and maize, suggesting parallel selection in different grass crops on this gene during domestication (Lin et al., 2012). In *S. italica*, the insertion of a *PIF/Harbinger* MITE close to the 3′ end of exon 2 led to the formation of a transcript that lacked exon 2. Exon 2 comprises 123 basepairs. Deletion of 41 amino acids in the SH1 protein in *S. italica* will almost certainly reduce or eliminate its activity. The same is true for proteins translated from transcripts in which introns 1, 2 and the MITE are retained. Analysis of resequencing data of *S. italica* (Jia et al., 2013) showed that 119 of the 200 accessions analyzed carried the MITE in *Sh1.* Of the 119 accessions, 75 were from China, and the remainder originated from other countries in Asia, Europe, and Africa. For the remaining 81 accessions, we could not establish presence/absence of the MITE because of the overall low depth of the resequencing data (average and median sequencing depth: 0.94 and 1.04, respectively). Importantly, none of the *S. italica* accessions tested were positive for the *S. viridis* control query sequence, supporting further that all *S. italica* germplasm carried the MITE in *Sh1*. Two additional *S. italica* lines, Ise-3 and Ise-5, that originated from India were also shown to carry the MITE in *Sh1* by PCR (**Supplementary Figure S1**).

Several hypotheses exist regarding the center of domestication of foxtail millet (reviewed by Diao and Jia, 2017). Some consider China the primary and possibly the only center of domestication for *Setaria*. Other studies advocate the existence of further independent centers of domestication in Europe and the region comprising central Asia, Pakistan, Afghanistan, and Northwest India. The presence of identical MITE insertions sites in *Sh1* in all 119 *S. italica* accessions for which sequence reads were available in the target region, irrespective of their country of origin, and the absence of this MITE in all *S. viridis* lines analyzed, strongly suggest a single center of domestication for foxtail millet. This is supported by the fact that alleles at SNP positions extending to at least 100 kb on either side of *Sh1* were close to fixation (99%) in our set of *S. italica* accessions (D. Chakraborty and K.M. Devos, unpublished data). The average frequency in this region of the *S. italica* SNP alleles in our set of *S. viridis* germplasm was 67%. The absence of the MITE in *Sh1* in the *S. viridis* germplasm analyzed may be due to (1) a low frequency of the low-shattering *Sh1* allele in wild populations, (2) insertion of the MITE early in the domestication process, or (3) a lack in our *S. viridis* collection of accessions from the center of domestication. Most of the resequencing data was for *S. viridis* accessions collected in the United States that belong to two major genetic subpopulations. Asian *S. viridis* germplasm largely forms a third subpopulation (Huang et al., 2014; Schröder et al., 2016)

It is unclear whether *qSH1* is the gene that underlies the shattering QTL on *Setaria* chromosome V. The presence of MITEs near genes can affect transcript levels, and both positive and negative effects have been reported (Naito et al., 2009; Lu et al., 2012; Han et al., 2013; Mao et al., 2015). It is therefore conceivable that the presence of either of the two MITEs in the 5′UTR of *qSH1* reduces expression of this transcription factor, leading to a strengthening of the abscission zone. In our analyses, transcript levels were lower in leaves of B100 than in A10, but no differences were observed in panicles. However, expression specifically in the abscission zone will need to be examined. In rice, *qSH1* expression was observed in both non-shattering and shattering lines in the shoot apical meristem during floral transition and in the anther regions, but only in shattering lines at the position of the abscission layer (Konishi et al., 2006). Although the coding sequence of the *qSH1* allele in *S. italica* acc. Yugu1 and B100 also differs from that in *S. viridis* accession A10 by the presence of a non-synonymous SNP, the lack of conservation of this region in different grass species (**Supplementary Figure S5**) suggests that this SNP is unlikely to affect *qSH1* gene function. An analysis of the presence of the two MITEs in *qSH1* across a worldwide collection of *S. italica* germplasm showed that 39% of Chinese germplasm (26 accessions) but only 7% (2) of accessions originating from countries other than China carried MITE 1. MITE 2 was predominantly present in *S. italica* worldwide. All *S. viridis* accessions tested lacked both MITEs. Genome-wide linkage disequilibrium in cultivated foxtail millet has been estimated at ∼100 kb (Jia et al., 2013), so our study could not differentiate between causal mutations and tightly linked variants. Of the three *S. italica* accessions that lacked both MITE 1 and MITE 2, two were from Hungary and one was from Turkey. The Turkish accession (Ci879) was listed as a “weedy foxtail” by Jia et al. (2013). Our study showed Ci879 to carry the MITE in *Sh1* so this accession was presumably low shattering. More precise phenotyping is necessary to determine the degree of shattering in *S. italica* lines that differ in the presence of the MITEs in *qSH1.*

## Conclusion

We identified *Sh1* on *Setaria* chromosome IX as the main target for reduced shattering during the domestication of foxtail millet. The variant allele, which carries a *PIF/Harbinger* MITE, was present in *S. italica* lines from around the world and absent from all *S. viridis* accessions tested, supporting the hypothesis of a single center of domestication for foxtail millet. The *S. italica* accession B100 carries at least one other gene for reduced shattering located on chromosome V. Structural variation is present at the *qSH1* locus between *S. italica* and *S. viridis*, and the presence of two MITEs in the 5′UTR of *qSH1* is strongly associated with cultivated genotypes. Complementation tests or knock-out experiments are needed to unambiguously demonstrate that the MITE insertions represent the causal mutations to shattering rather than linked variants.

## Author Contributions

SO participated in the experimental design and QTL analysis, conducted the comparative analysis, and drafted the manuscript. SC did the *qSH1* transcript analysis, conducted the PCR to confirm the presence of the MITEs in *Sh1* and *qSH1*, and analyzed the RNA-Seq reads in the *Sh1* region. DC conducted the bioinformatic analysis of the presence of the MITEs in the *S. viridis* and *S. italica* germplasm. LM-B did the experimental *Sh1* transcript analysis. MT and OP conducted the phenotyping of the F_2_ population. XW conducted the phenotyping of the RIL population. BB conducted the QTL analyses. KD designed the study, participated in all aspects of data analysis and data interpretation, and wrote the manuscript. All authors critically read and approved the manuscript.

## Conflict of Interest Statement

The authors declare that the research was conducted in the absence of any commercial or financial relationships that could be construed as a potential conflict of interest.

## Footnotes

1 http://phytozome.jgi.doe.gov

2 phytozome.jgi.doe.gov

3 genome.jgi.doe.gov

## Supplementary Material

FIGURE S1 Multi-sequence alignment of the *Sh1* alleles and transcripts in *S. viridis* accession A10 and *S. italica* accessions Yugu1, B100, Ise-3 and Ise-5. Genomic and cDNA sequences are indicated with “genomic” and “cDNA,” respectively, in the sequence name. Intron sequences have a white background, exon sequences have brown (A), yellow (C), green (T), and purple (G) backgrounds, and MITE sequences have green (A), orange (C), blue (T), and red (G) backgrounds.

FIGURE S2 Insertion of **(A)** MITE 1 and **(B)** MITE 2 in the 5′UTR of *qSH1* was accompanied or followed by rearrangements. The MITE sequence is delineated by “|”. Deletions are in red, insertions in green. [ ] indicates SSR units. Microhomology flanking the deletion is highlighted in yellow.

FIGURE S3 Multi-sequence alignment of the *qSH1* alleles in *S. viridis* accession A10, and *S. italica* accessions B100 and Yugu1. MITE sequences have green (A), orange (C), blue (T), and red (G) backgrounds. The “ATG” start codon has a white background.

FIGURE S4 Alignment of RNA-Seq reads against *Sh1* genomic sequence. **(A)** A10 reads (SRX875196) against Sevir.9G153200 (only the read coverage is shown) and **(B)** Yugu1 reads (SRX2832831) against Seita.9G154300 (both read coverage and individual read alignments are shown). Exon locations are indicated with red boxes. The MITE location in Seita9G.154300 is shown in blue.

FIGURE S5 Multi-sequence alignment at the protein level across different grass species of the *qSH1* region carrying the non-synonymous SNP that differentiates *S. italica* from *S. viridis* (position 31 in this alignment).

TABLE S1 Genotypic and phenotypic data for the B100 × A10 F_2:3_ and RIL populations.

TABLE S2 Semi-quantitative RT-PCR of *qSH1* in leaves and panicles of A10 and B100.

TABLE S3 Presence/absence of MITEs 1 and 2 in *qSH1* in *S. italica* germplasm.
