# Supplementary material for: QTL Mapping Combined With Comparative Analyses Identified Candidate Genes for Reduced Shattering in Setaria italica
Source: Front Plant Sci. 2018 Jul 19;9:918. doi: 10.3389/fpls.2018.00918 (PMC6060267; doi:10.3389/fpls.2018.00918)
Supplement: FIGURE S2 — Insertion of (A) MITE 1 and (B) MITE 2 in the 5′UTR of qSH1 was accompanied or followed by rearrangements. The MITE sequence is delineated by “|”. Deletions are in red, insertions in green. [ ] indicates SSR units. Microhomology flanking the deletion is highlighted in yellow. [file Image_2.PDF]

A. Insertion of MITE 1 followed or accompanied by a deletion

CTAGCTCTAGCTACCCTCTTTTCCGCCAGGCCTCCGGTCCTCTCTCG|AGGGGGTGTTTGGGAAACACCT
GTTAAAGTTTAACACCTATCACATCGGATGTTTGGATGCTAATTAGGAGGATTAAACATGAGCTAATTAT
AAAACTAATTGCACAACCTTGTGCTAATTCGCGAGACGAATCTATTAAGTCTAATTAATCCATCATTAGC
AAATGGTTACTGTAGCACCACATTGTCAAATCATGGACTAATTANGCTTAATAGATTCGTCTCGCGAATT
ATACTCCATCCGTGCAATTAGTTTTGTAATTAGCTTATGTTTAATACTCCTAATTAGGATCCAAACATCC
GATGTGATGGGTGTTAAACTTTAACAGGTGTTTCCCAAACACCCCCT|GATGAGTTCTCGGTCTCGGCTT
TGCCGAAAGAAAGGCACCCGGA

B. Insertion of MITE 2 followed or accompanied by rearrangements

GGGCAGCAGGACCGCCGCTCGGAAAGTGCGGAGCCGTTTCCCCTCTAGCCTCTCTACTT|GGCCGTGTTT
AGTTTGGCGAATTTGGGGGTGCTAAATTACAGCACTGTAGCACACTGTAGCGTTTCGTTTGTATTTGTGA
ATTATTGTCCAAATATTGACTAATTAGGCTCAAAAGATTCGTCTCGCAAAGTACAACAAAACTGTGCAAT
TAGTTTTTAATTTCATCTACATTTAGTACTCCATGCATGTACCGCAAGTTTGATGTGATGGGGAATCTTC
TTTTTGCATAGTGTCAAAGTTGGGAGTTGGGAGTAACTAAACATGG|GGGCCAGGT[GCCCAC][GCCCA
C][GCCCAC][GCCCAC][GCCCAC]GCCTCGGGCGCTCTCCTCCAGGGGGC

Supplementary Figure S2: Insertion of (A) MITE 1 and (B) MITE 2 in the 5’ UTR of *qSH1* was accompanied or followed by rearrangements. The MITE sequence is delineated by |. Deletions are in red, insertions in green. [ ] indicate SSR units. Microhomology flanking the deletion is highlighted in yellow.
