# Supplementary figures and images for: QTL Mapping Combined With Comparative Analyses Identified Candidate Genes for Reduced Shattering in Setaria italica
Source: Front Plant Sci. 2018 Jul 19;9:918. doi: 10.3389/fpls.2018.00918 (PMC6060267; doi:10.3389/fpls.2018.00918)

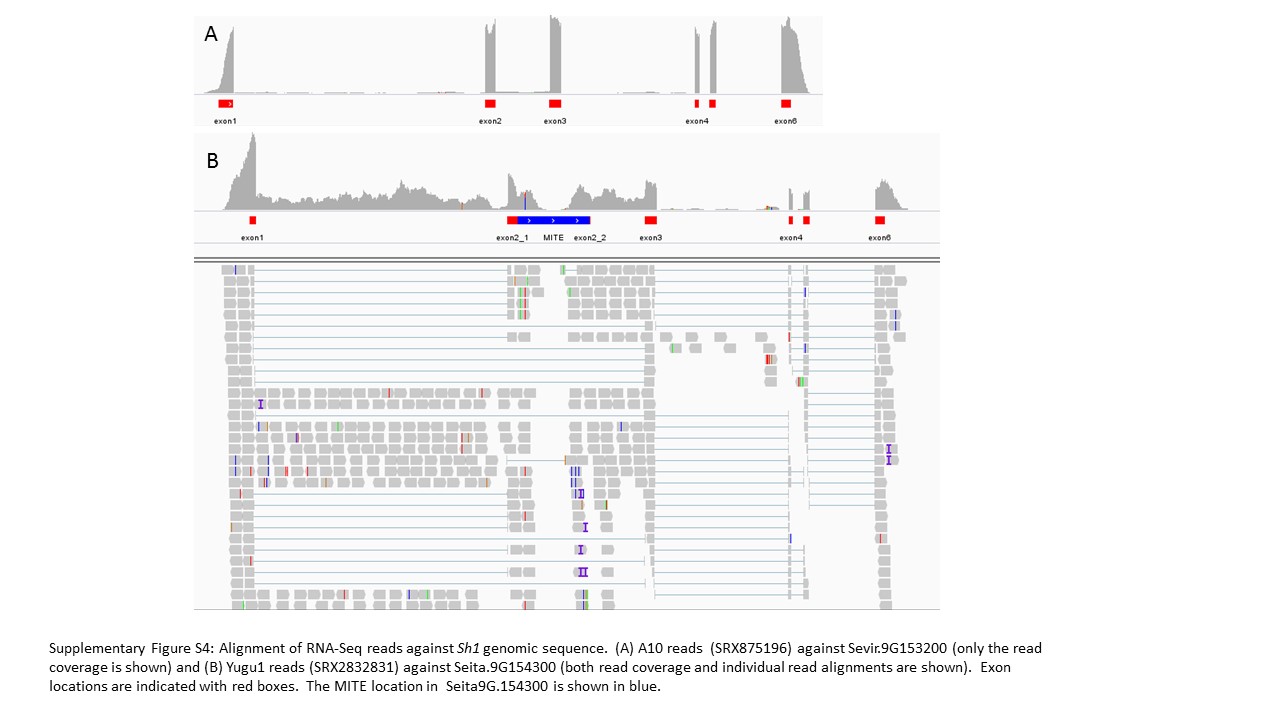

Supplement: FIGURE S4 — Alignment of RNA-Seq reads against Sh1 genomic sequence. (A) A10 reads (SRX875196) against Sevir.9G153200 (only the read coverage is shown) and (B) Yugu1 reads (SRX2832831) against Seita.9G154300 (both read coverage and individual read alignments are shown). Exon locations are indicated with red boxes. The MITE location in Seita9G.154300 is shown in blue. [file Image_4.jpg]

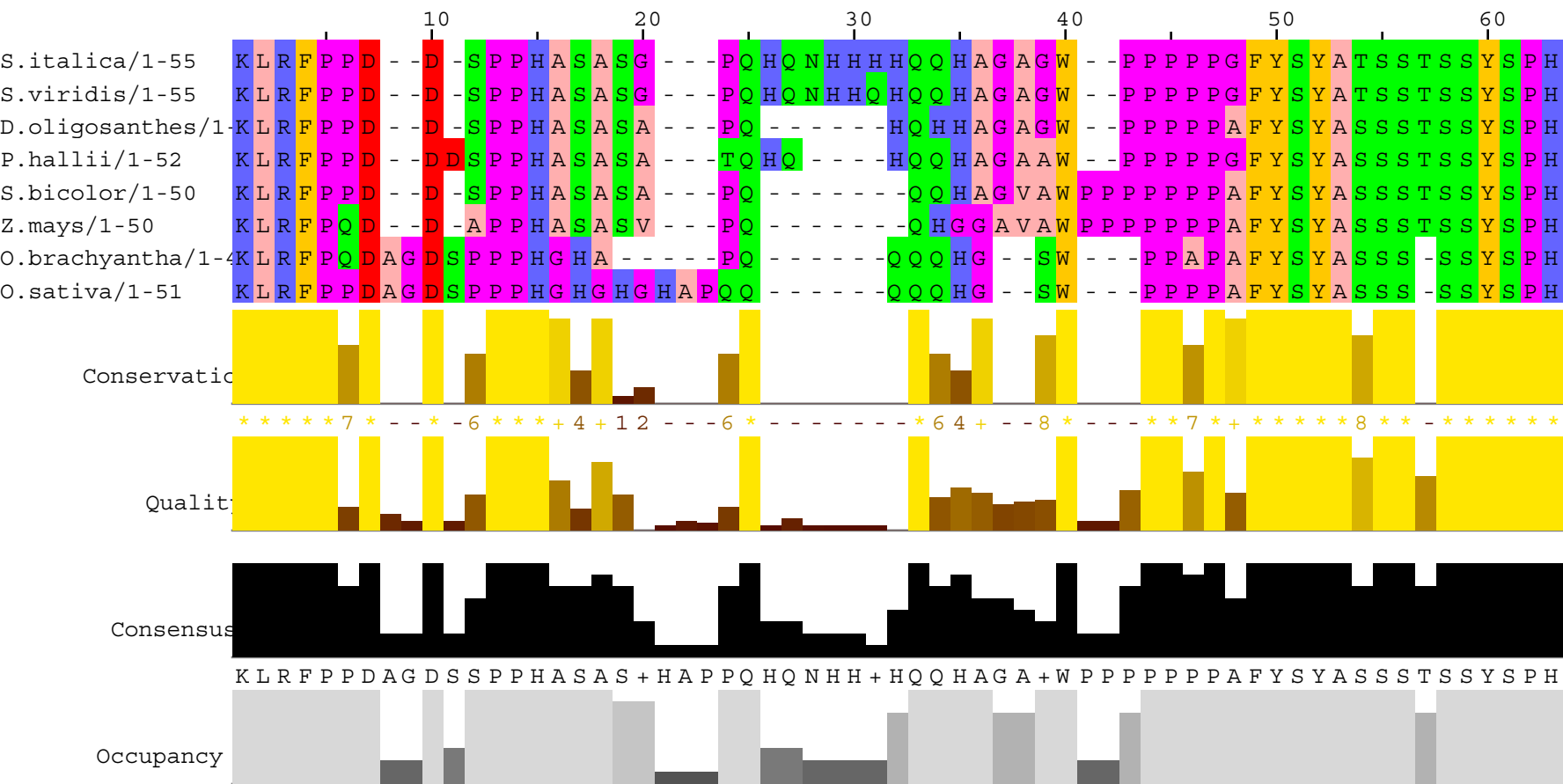

Supplement: FIGURE S5 — Multi-sequence alignment at the protein level across different grass species of the qSH1 region carrying the non-synonymous SNP that differentiates S. italica from S. viridis (position 31 in this alignment). [file Image_5.PDF]
