# Supplementary material for: QTL Mapping Combined With Comparative Analyses Identified Candidate Genes for Reduced Shattering in Setaria italica
Source: Front Plant Sci. 2018 Jul 19;9:918. doi: 10.3389/fpls.2018.00918 (PMC6060267; doi:10.3389/fpls.2018.00918)
Supplement: TABLE S2 — Semi-quantitative RT-PCR of qSH1 in leaves and panicles of A10 and B100. [file Table_2.docx]

Supplementary Table S2: Semi-quantitative RT-PCR of *qSH1* in leaves and panicles of A10 and B100

|  | *qSH/Actin* ratio | | |
| --- | --- | --- | --- |
| Plant ID^1^ | Techn. Rep. 1 | Techn. Rep. 2 | Techn. Rep3 |
| Leaves | | | |
| A10-1 | 0.542 | 0.444 | 0.647 |
| A10-3 | 0.687 | 0.510 | 0.950 |
| A10-4 | 0.584 | 0.433 | 0.416 |
| B100-1 | 0.197 | 0.280 | 0.172 |
| B100-3 | 0.148 | 0.208 | 0.084 |
| B100-4 | 0.291 | 0.345 | 0.303 |
| Panicles 40 days after heading | | | |
| A10-5 | 1.177 | 0.900 | 1.254 |
| A10-6 | 2.020 | 1.629 | 3.306 |
| A10-7 | 1.438 | 1.388 | 2.767 |
| B100-5 | 1.255 | 1.098 | 1.840 |
| B100-6 | 1.057 | 0.870 | 1.321 |
| B100-7 | 1.157 | 0.622 | 1.795 |

^1^ Numbers after the accession name indicate different plants of the same accession
