# Supplementary material for: QTL Mapping Combined With Comparative Analyses Identified Candidate Genes for Reduced Shattering in Setaria italica
Source: Front Plant Sci. 2018 Jul 19;9:918. doi: 10.3389/fpls.2018.00918 (PMC6060267; doi:10.3389/fpls.2018.00918)

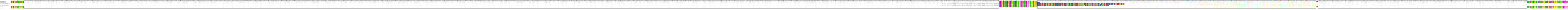

Supplement: FIGURE S1 — Multi-sequence alignment of the Sh1 alleles and transcripts in S. viridis accession A10 and S. italica accessions Yugu1, B100, Ise-3 and Ise-5. Genomic and cDNA sequences are indicated with “genomic” and “cDNA,” respectively, in the sequence name. Intron sequences have a white background, exon sequences have brown (A), yellow (C), green (T), and purple (G) backgrounds, and MITE sequences have green (A), orange (C), blue (T), and red (G) backgrounds. [file Image_1.PDF]
