# Supplementary material for: QTL Mapping Combined With Comparative Analyses Identified Candidate Genes for Reduced Shattering in Setaria italica
Source: Front Plant Sci. 2018 Jul 19;9:918. doi: 10.3389/fpls.2018.00918 (PMC6060267; doi:10.3389/fpls.2018.00918)

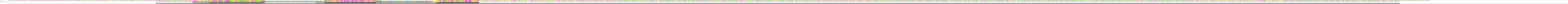

Supplement: FIGURE S3 — Multi-sequence alignment of the qSH1 alleles in S. viridis accession A10, and S. italica accessions B100 and Yugu1. MITE sequences have green (A), orange (C), blue (T), and red (G) backgrounds. The “ATG” start codon has a white background. [file Image_3.pdf]
